# Colony Formation, Migratory, and Differentiation Characteristics of Multipotential Stromal Cells (MSCs) from “Clinically Accessible” Human Periosteum Compared to Donor-Matched Bone Marrow MSCs

**DOI:** 10.1155/2019/6074245

**Published:** 2019-11-21

**Authors:** Heather E. Owston, Payal Ganguly, Giuseppe Tronci, Stephen J. Russell, Peter V. Giannoudis, Elena A. Jones

**Affiliations:** ^1^Institute of Medical and Biological Engineering, University of Leeds, Leeds, UK; ^2^Clothworkers' Centre for Textile Materials Innovation for Healthcare, University of Leeds, UK; ^3^Leeds Institute of Rheumatic and Musculoskeletal Medicine, University of Leeds, Leeds, UK

## Abstract

Periosteum is vital for fracture healing, as a highly vascular and multipotential stromal cell- (MSC-) rich tissue. During surgical bone reconstruction, small fragments of periosteum can be “clinically accessible,” yet periosteum is currently not ultilised, unlike autologous bone marrow (BM) aspirate. This study is aimed at comparing human periosteum and donor-matched iliac crest BM MSC content and characterising MSCs in terms of colony formation, growth kinetics, phenotype, cell migration patterns, and trilineage differentiation capacity. “Clinically accessible” periosteum had an intact outer fibrous layer, containing CD271+ candidate MSCs located perivasculary; the inner cambium was rarely present. Following enzymatic release of cells, periosteum formed significantly smaller fibroblastic colonies compared to BM (6.1 mm^2^ vs. 15.5 mm^2^, *n* = 4, *P* = 0.0006). Periosteal colonies were more homogenous in size (range 2-30 mm^2^ vs. 2-54 mm^2^) and on average 2500-fold more frequent (2.0% vs. 0.0008%, *n* = 10, *P* = 0.004) relative to total viable cells. When expanded *in vitro*, similar growth rates up to passage 0 (P0) were seen (1.8 population doublings (PDs) per day (periosteum), 1.6 PDs per day (BM)); however, subsequently BM MSCs proliferated significantly slower by P4 (4.3 PDs per day (periosteum) vs. 9.3 PDs per day (BM), *n* = 9, *P* = 0.02). In early culture, periosteum cells were less migratory at slower speeds than BM cells. Both MSC types exhibited MSC phenotype and trilineage differentiation capacity; however, periosteum MSCs showed significantly lower (2.7-fold) adipogenic potential based on Nile red : DAPI ratios with reduced expression of adipogenesis-related transcripts *PPAR-γ*. Altogether, these data revealed that “clinically accessible” periosteal samples represent a consistently rich source of highly proliferative MSCs compared to donor-matched BM, which importantly show similar osteochondral capacity and lower adipogenic potential. Live cell tracking allowed determination of unique morphological and migration characteristics of periosteal MSCs that can be used for the development of novel bone graft substitutes to be preferentially repopulated by these cells.

## 1. Introduction

Fracture nonunion represents a significant clinical challenge. The rate of fracture nonunion is reported to be between 2 and 10% of all fractures, dependent on fracture type, location, and patient demographics [1, 2]. Its treatment is not only costly to healthcare providers [3], but importantly to the patients through loss of earnings and high rates of depression, all of which will have a detrimental effect on quality of life [4]. Therefore, improvements to standard-of-care treatments of bone fracture are needed and have been recently developed based on the diamond concept framework [5], which draws attention to the importance of ensuring the presence of all: osteogenic cells, osteoconductive scaffolds, osteoinductive growth factors and a stable mechanical environment at the site of healing [5]. Most commonly, osteogenic cells are delivered to the fracture site in the form of bone marrow aspirate (BMA), BMA concentrate (BMAC), or combining BMAC with grafting material to fill larger defects to aid with fracture repair [6–10].

The critically important role of periosteum during bone fracture healing is well established. Following fracture periosteum reacts by thickening and entering into a proliferative state [11–13]. Periosteal-derived progenitor cells migrate into the fracture haematoma early on, where they differentiate into osteoblasts or chondrocytes, directly contributing to bone regeneration [12]. Periosteal stripping or damage during fracture results in decreased callus formation or delayed union [13–16]. However, despite the known critical impact of periosteum during fracture repair, the use of periosteal-derived cells for surgical repair of fractures, nonunions, or critical size bone defects remains unexploited.

Periosteum lines the outer aspect of most bones, contributing to appositional growth during childhood and adolescence as well as providing the vascular supply to bone [14]. This tissue, although thin, forms two distinct layers, the bone lining inner cambium layer being highly cellular and containing osteogenic progenitors, while the outer fibrous layer, predominantly consisting of collagen, is highly vascular but not particularly cellular in comparison [12, 17]. The cambium layer is tightly attached to the underlying bone by Sharpey's Fibres; thus, removal of periosteum with the cambium layer attached is difficult [18] and is furthermore considered inappropriate by the surgeons as potentially compromising the repair process. However, “clinically accessible” samples of periosteum, likely to be fibrous in nature, can be obtained as by-products of surgical debridement of the fracture site, but their osteogenic progenitor cell content has not yet been explored before.

The purpose of this study was to investigate “clinically accessible” samples of human periosteum for the presence of osteogenic progenitor multipotential stromal cells (MSCs) and to compare their functional capacities with iliac crest BMA samples from the same donors.

## 2. Materials and Methods

### 2.1. Tissue Collection and Processing

Donor-matched samples of periosteum and BMA were obtained from 12 patients treated for fracture nonunion (median age 49 years, range 17-80) at the Trauma Orthopaedic Unit at Leeds General Infirmary, Leeds, UK. Periosteum (approximately 50 mm^2^, 0.3 g) was harvested from near to the site of fracture using a scalpel, and BMA (volume range 16-60 mL) was aspirated from the anterior iliac crest. Iliac crest trabecular bone (approximately 1 cm^3^) was collected from three patients, undergoing autograft procurement. Ethical approval was granted by the National Research Ethics Committee–Leeds East, with ethical approval number 06/Q1206/127 and informed written consent given by all patients.

For extraction of periosteum- and BMA-resident nucleated cells, BMA underwent processing with ammonium chloride (Stem Cell Technologies) to lyse red blood cells. Briefly, 4 mL of ammonium chloride solution was added per 1 mL of BMA and incubated for 10 mins on ice. Cells were concentrated via centrifugation (650 g, 5 mins). Periosteum samples were digested in collagenase (600 U/mL, Stem Cell Technologies) at a ratio of 0.1 g of tissue to 0.5 mL of collagenase, for 4 h, incubated at 37°C, 5% CO_2_. After digestion, the cell suspension was passed through a 70 *μ*m cell strainer to remove large debris; the cell suspension was concentrated via centrifugation (650 g, 5 mins). Cells were used directly for *in vitro* assays, as described in the subsequent sections.

### 2.2. Histology of Periosteum

Human periosteum and iliac crest bone samples were fixed in 3.75% formaldehyde for a week, and iliac crest bone was next decalcified using 0.1 M EDTA. Subsequently, samples were embedded in paraffin wax, and 5 *μ*m thick slides were produced (SuperFrost Plus slides). Slides were dewaxed and hydrated using standard methods, followed by staining for haematoxylin and eosin and picro sirius red. Samples were also stained for CD271 (1 : 20, NGFRS, Abcam), the well-recognised marker of bone-resident MSCs [19]. Antigen retrieval was carried out using 10 mM/L citrate buffer, and immunohistochemistry was carried out using the Dako EnVision kit (Dako), as per the manufacturer's guidelines. Post staining, slides were dehydrated and cleared with xylene and covered with a coverslip, using DPX (Sigma). Slides were imaged using light microscopy (AxioCam MRc5, Zeiss) and under a polarised light.

### 2.3. Colony-Forming Fibroblast (CFU-F) Assay

CFU-F assays were used to quantify colony-forming cells in BMA and periosteum digest samples and performed as previously described [20, 21]. After 14 days of culture in StemMACS MSC expansion media (Miltenyi Biotec, supplemented with 1% penicillin streptomycin), duplicate dishes were fixed, stained [20], and scanned. Colonies were counted and colony surface area was quantified using ImageJ [22]. The frequency distribution of colony surface area was plotted using Prism. The spread of colony surface area distribution data was quantified using “full width at half maximum” (FWHM) values [23].

### 2.4. MSC Expansion and Surface Marker Characterisation

Following initial processing, nucleated cells from BMA and periosteum were plated at a density of 4 × 10^5^ per cm^2^ and 1 × 10^4^ per cm^2^, respectively, and incubated at 37°C, 5% CO_2_ in StemMACS media. After 48 hours, media were replaced and cells were cultured until 60-80% confluence with biweekly half medium changes; flasks were trypsinised and reseeded at cell densities of 1.25 × 10^5^ for periosteum [24] and 2.5 × 10^5^ for BM [25], this was repeated until passage 6. Population doubling (PD) up to passage 0 (<P0) and after passage 0 (>P0) was calculated as follows: PD (<P0) = log_2_(cell count at passage 0/seeded number of CFU − F) and PD (>P0) = log_2_(cell count at passage/seeded cell count) as per Churchman et al. [26].

To confirm the MSC nature of expanded cells, flow cytometry of donor-matched BM and periosteum cultures was carried out using antibodies chosen in line with the International Society for Cellular Therapies (ISCT) approved panel (Supplementary [Supplementary-material supplementary-material-1]) [27], as previously described by [28]. In brief, trypsinised cells were resuspended in blocking buffer (10% mouse serum, 1% human IgG in FACS buffer (0.1% BSA, 0.01% sodium azide, 0.5 M EDTA in PBS)) for 15 mins at RT and stained with antibodies (Supplementary [Supplementary-material supplementary-material-1]) for 30 mins on ice. Following one wash in FACS buffer, cells were resuspended in 500 *μ*L 4′,6-diamidino-2-phenylindole (DAPI) buffer ready to be analysed by an LSRII flow cytometer (BD Pharmingen). Data was analysed using FACS DIVA software.

### 2.5. *In Vitro* Trilineage Differentiation Assays

Cultured cells (passage 1-3) from donor-matched periosteum and BM underwent trilineage differentiation assessment; OsteoDIFF, AdipoDIFF, and ChondroDIFF media (Miltenyi Biotec) were used to induce differentiation. For osteogenesis (*n* = 7 donors), 2600 cells/cm^2^ were plated onto 7 replicate flat bottom wells and incubated at 37°C, 5% CO_2_ in OsteoDIFF for 2 or 3 weeks, with biweekly, half medium changes. At two weeks, alkaline phosphatase (ALP) activity was detected using fast blue. At three weeks, calcium deposition was stained with alizarin red [29] and calcium content was measured, as previously described [28]. For three donors, an extra well was set up to measure DNA content, cells were lysed in 200 *μ*L of 0.1% Triton-X 100, and DNA content was quantified using a PicoGreen Assay (Thermo Scientific) [30].

Adipogenesis assays were seeded at 4200 cells/cm^2^ into 5 replicate flat bottomed well plates, incubated at 37°C, 5% CO_2_, and half medium (AdipoDIFF) changes were carried out biweekly for 3 weeks. After 3 weeks, plates were fixed with 3.75% formaldehyde and stained with either oil red (*n* = 6 donors) or Nile red and DAPI (*n* = 3 donors) [31], the latter of which was quantified using a fluorescent plate reader (Berthold) and Nile red absorbance levels were normalised to DAPI absorbance levels to establish Nile red/DAPI ratios [31].

Chondrogenic assays (*n* = 5 donors) were carried out in 5 replicate 1.5 mL screw cap Eppendorf tubes; 2.5 × 10^5^ cells were added to each tube and centrifuged (800 g, 5 mins) to create a pellet culture and was resuspended in ChondroDIFF media. Tubes were placed in an incubator at 37°C, 5% CO_2_ for 3 weeks, where half medium changes were made three times a week. After 3 weeks, 2 pellets were snap frozen in OCT, cut using a cryostat (Leica Biosystems), and dried onto histology slides where toluidine blue was used to stain GAGs. The remaining three pellets were digested in papain digest buffer at 65°C overnight, and the GAG content was quantified using a sulphated GAG assay kit (Blyscan), as previously described [28].

Extra wells or pellets were set up for each trilineage differentiation assay for 3 donors to allow quantification of change expression of key lineage markers following differentiation induction. Cells were lysed and RNA isolated using a Single Cell RNA Purification Kit (Norgen) and on-column DNase (Applied Biosystems) treatment. cDNA was produced using a High-Capacity cDNA Reverse Transcription Kit (Applied Biosystems) for use with TaqMan assays: osteogenesis markers—runt-related transcription factor 2 (RUNX2) and bone gamma-carboxyglutamate (gla) protein (BGLAP); chondrogenesis markers—collagen, type 2, alpha 1 (*COL2A1*) and SRY- (sex-determining region Y-) box 9 (*Sox9*); and adipogenesis markers—fatty acid-binding protein 4 (*FABP4*) and peroxisome proliferator-activated receptor-gamma (*PPAR-γ*). The real time-PCR (RT-PCR) reaction was run using a QuantStudio™ 7 Flex Real-Time PCR System and SDS software, recording the fluorescence in real time. Analysis was carried out using the 2^-*Δ*(Ct)^ method, to calculate normalised gene expression (normalised to hypoxanthine phosphoribosyltransferase 1 (HPRT1) expression) [32].

### 2.6. Live Holographic Imaging

P0 vials of donor-matched (male, 17) BM and periosteum MSCs were seeded in duplicate (250 cells per well) onto a Lumox® multiwell 24-well cell culture plate (Sarstedt). The cultures were grown for 3 days at 37°C, 5% CO_2_ in StemMACS media. On day 3, following a half medium change, the plate lid was replaced with PHI HoloLids™ imaging covers (PHI, phase holographic imaging) (sterilised in 70% EtOH, 10 mins). The plate was placed onto the xy motorised stage of a HoloMonitor M4 Microscope, set up inside an incubator. Using the Hstudio software, three coordinates in each well were manually focused and set to automatically image every hour for 2 days.

Post acquisition, the Hstudio software applied a “mask” to the images, where a threshold was set to distinguish cells from the bottom of the well, allowing for automatic cell identification. Automatic cell number assignment allowed for individual cell tracking over time, which was checked manually. All outputs were exported into Excel files and plotted using Prism.

### 2.7. Statistical Analysis

To compare donor-matched periosteum and BM samples, statistical analysis was carried out using appropriate paired tests (Wilcoxon signed-rank test or paired *t* test depending on data distributions), where *P* < 0.05 was considered significant. Phase holographic imaging data sets were compared using an unpaired Student's *t*-test. The exact tests are specified in the figure legends, and the data is presented mean ± standard deviation.

## 3. Results

### 3.1. Location of CD271+ Candidate MSCs in Human Periosteum and Iliac Crest Bone

**Figure 1:**
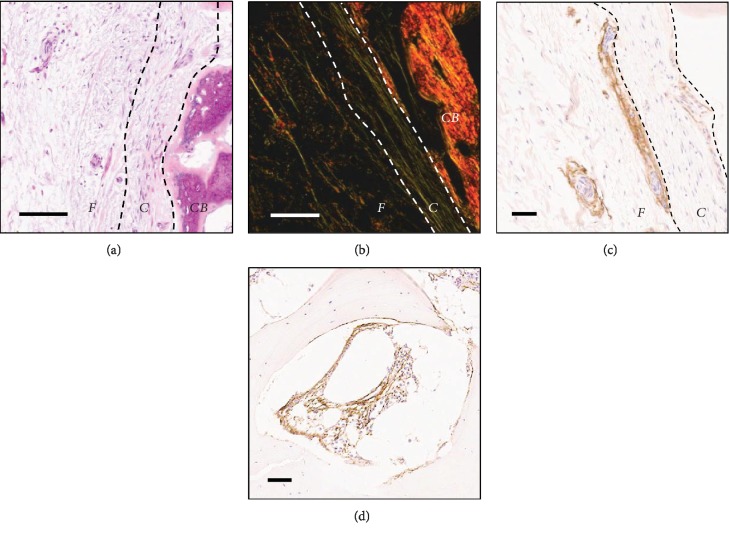
Histological staining of human periosteum samples, harvested close to a fracture site. (a) H&E staining of periosteum sample showing the cellular inner cambium layer (*C*), the lining cortical bone (*CB*), and the outer fibrous layer (*F*). (b) Picro sirius red, imaged using polarised light, showed collagen as the dominant feature of periosteum. CD271 candidate MSC marker immunohistochemistry staining was carried out on (c) periosteum, where staining was shown in the cambium layer and surrounding blood vessels throughout the fibrous layer and (d) iliac crest bone samples, where staining was within the bone marrow and lining the bone.

The architecture of human samples of periosteum (*n* = 5), taken from the femur and humerus (*n* = 1), approximately 5 cm from a nonunion fracture site (within the surgical opening) was assessed. The mean donor age was 51.8 ± 24.6 years (range, 23-80 years), and periosteum samples were harvested 50 ± 37 weeks following initial injury, with each patient having undergone 0-2 previous orthopaedic surgeries (Table 1). In all the samples, apart from one (male, 47) (Figures 1(a)–1(c)), no cambium layer could be seen, reflecting the preferential harvesting of periosteum fibrous layer, as expected. When an intact cambium layer was seen (Figures 1(a) and 1(b)), it was shown to be attached to the underlying bone. Here, the periosteum could be clearly split into the bone lining cambium layer and the muscle facing fibrous layer.

To investigate the location of candidate MSCs throughout the periosteum, CD271 staining was carried out. CD271 positivity was found throughout both layers of the periosteum, localised to the outer edge of blood vessels (Figure 1(c)). CD271 positivity was also seen in the BM cavities of the control iliac crest bone as expected (Figure 1(d)) [33].

### 3.2. Colony Formation and Growth Kinetics of Periosteum-Derived Cultures

**Figure 2:**
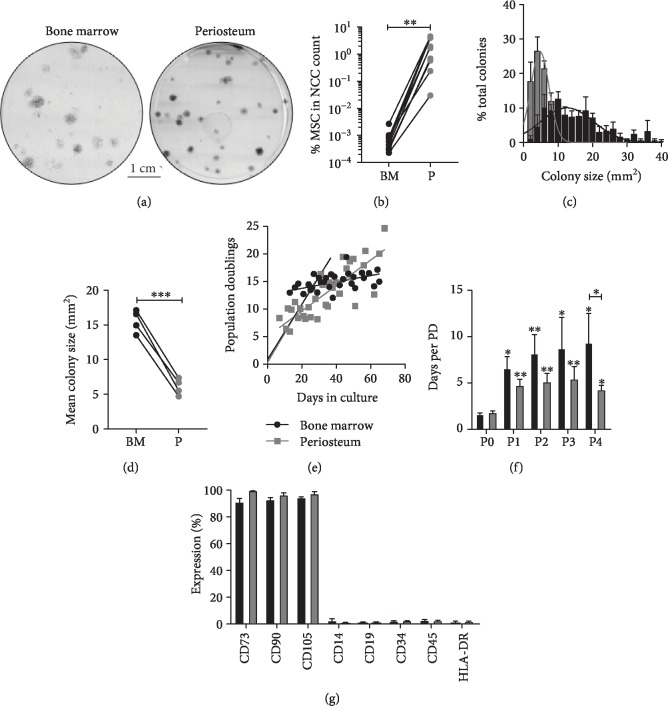
Quantification of MSC content, colony formation, and *in vitro* proliferation of periosteum and bone marrow-derived MSCs. (a) Colonies formed by MSCs during CFU-F assays. (b) Percentage MSCs in nucleated cell count. (c) Quantification of colony surface area (*n* = 4) distribution (Gaussian distribution). (d) Mean colony size (*n* = 4). (e) *In vitro* proliferation of MSCs, calculated by cumulative population doublings, showing two distinct growth curves prior to passage (<P0) and post passage (>P0). (f) Days per population doubling compared from P0 to P4. (g) Flow cytometry histograms for positive and negative MSC markers. Statistical analysis was carried out, Wilcoxon signed-rank test ^∗∗^*P* = 0.004 (a), paired Student's *t*-test ^∗∗∗^*P* = 0.0006 (d), Kruskal-Wallis test (comparison of P0 to P1-P4) ^∗^*P* < 0.02, ^∗∗^*P* < 0.001 (f), and Wilcoxon signed-rank test (comparison of bone marrow to periosteum) ^∗^*P* < 0.05 (f). BM: bone marrow (black), P: periosteum (grey), NCC: nucleated cell, PD: population doubling.

CFU-F assays were carried out to quantify colony formation as a measure of MSC frequency in donor-matched iliac crest BM and periosteum samples (*n* = 10) (Figures 2(a) and 2(b)). The mean donor age was 46.3 ± 17.3 years (range, 17-74 years), periosteum was harvested mainly from the femur (*n* = 7), but also the tibia (*n* = 2) and humerus (*n* = 1), all from nonunion cases, 70 ± 47 weeks following initial injury, and patients had undergone 0-2 previous orthopaedic surgeries.

In the BM, CFU-F frequency in relation to total nucleated cells was on average 0.0008 ± 0.0002% consistent with previous studies [21, 34]. In donor-matched periosteum digests, CFU-F frequency was significantly (*P* = 0.004, Wilcoxon signed-rank test) higher (2500-fold) (2.0 ± 0.6%) (Figure 2(b)).

In order to assess the visual differences in colony formation (Figure 2(a)), colony surface area was quantified, from donor-matched dishes containing more than 20 colonies (*n* = 4). Periosteum colonies were more homogenous, with a smaller size distribution compared to BM colonies (Figure 2(c)), which were more heterogeneous and larger. Periosteum colonies were significantly smaller at 6.1 ± 0.6 mm^2^ (range, 2.0-29.6 mm^2^) compared to BM colonies at 15.5 ± 0.8 mm^2^ (range, 2.1-54.3 mm^2^) (paired Student's *t*-test, *P* = 0.0006) (Figure 2(d)). Of particular interest was that BM had a subset of larger colonies > 30 mm^2^, accounting for 6.4 ± 2.2% of all BM colonies, which were not seen in periosteum samples.

Growth kinetics of donor-matched BM and periosteum cultures was next investigated, and two clear growth patterns were noticeable (Figures 2(e) and 2(f)) and thus split before P0 (<P0) and after P0 (>P0) for the analysis. Growth rates measured as PDs per day for <P0 cultures were similar between BM (1.6 ± 0.1 days per PD) and periosteum (1.8 ± 0.2 days per PD), also shown through similar slope gradient (0.50 (BM) and 0.52 (P)) following linear regression analysis (*r*^2^ values, 0.87 (BM) and 0.91 (periosteum)) (Figure 2(e)). However, differences could be seen following quantification of number of PDs at P0; BM cultures were started with a lower number of MSCs compared to periosteum (Figure 2(b)) and therefore went through significantly higher number of PDs (BM: 13.3 ± 0.5 PDs at P0, periosteum: 8.5 ± 1.1 PDs at P0, Wilcoxon signed-rank test, *P* = 0.02).

Following the first passage, the PD rate significantly slowed (P1-P4) for both culture types (Figures 2(e) and 2(f)), by P4 proliferation rates were reduced to 9.3 ± 3.2 days per PD (BM) and 4.3 ± 0.5 days per PD (periosteum) (Figure 2(f)). The ISCT MSC phenotype was tested on donor-matched cultures [27] at passage 4, and both culture types were >91% positive for MSC markers CD73 (ecto-5′-NT), CD90 (Thy1), and CD105 (Endoglin) and <3% positive for hematopoietic lineage markers: CD14 (monocyte differentiation antigen), CD19 (B-lymphocyte antigen), CD34 (haematopoietic progenitor cell antigen), CD45 (haematopoietic cell marker), and HLA-DR (MHC class II cell surface receptor) (Figure 2(g)).

Overall, these data revealed high CFU-F content and a more homogenous nature of colony-forming cells in “clinically accessible” periosteal samples, compared to donor-matched BM samples. Additionally, in our chosen experimental conditions, periosteum MSC cultures had achieved lower cumulative PDs than their BM counterparts at the same passage.

### 3.3. Live Cell Tracking of Periosteum and Bone Marrow MSC Cultures

To explore if the observed differences seen in colony size between periosteum and BM MSCs could be due to different cell migration patterns, live cell imaging and tracking of cell movement over time was carried out using holographic imaging. Thawed P0 cell cultures (previously grown in culture for 13 days, 6.6 PD (periosteum) and 13 PD (BM)) were tracked from one donor (male, 17). Thawed freshly digested periosteum cultures were grown and shown to be not significant to their P0 counterparts (data not shown); therefore, P0 cultures were taken as surrogate to represent MSC migration for up to 2 weeks in culture (the end time point of a CFU-F assay).

**Figure 3:**
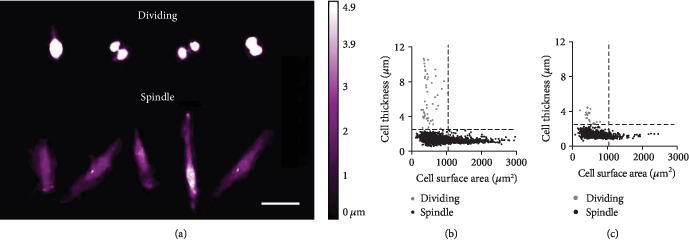
Distinct morphology types of MSCs described as “dividing” and “spindle” shaped. (a) Four individual cells in the process of cell division (top row) and five individual “spindle-” shaped cells, the classical MSC phenotype (bottom row). All quantified cells were plotted cell surface area vs. cell thickness to show the two different morphologies for (b) periosteum and (c) bone marrow cultures. Scale bar represents 50 *μ*m. Dividing cells (grey) and spindle-shaped cells (black). Separation of cell types at *y* = 2.5 *μ*m and *x* = 1100 *μ*m^2^.

Following thresholding of individual images and identification of cells, the morphology of the cells within each culture could be quantified and compared. There were two differing cell morphologies within both MSC populations. The first of which was “dividing” cells (Figure 3(a)), with high average cell thickness (>2.5 *μ*m) but a small cell surface area (<1100 *μ*m^2^), reflecting a spherical shape just prior to cell division, which made up 2.05% (periosteum) and 2.12% (BM) of all cells (Figures 3(b) and 3(c)). The second, which formed the majority (97.95%: periosteum, 97.88%: BM), was “spindle-” shaped cells (Figure 3(a)), representing the classical MSC morphology, with an average cell thickness of 0.6-2.5 *μ*m and cell surface area ranging from 79 to 2965 *μ*m^2^ (Table 2).

With respect to the “dividing” cells, the average thickness of periosteum cells (6.1 ± 2.5 *μ*m) was significantly higher (unpaired Student's *t*-test, *P* < 0.0001) than BM cells (3.4 ± 0.6 *μ*m); however, cell surface area was similar (Table 2). In addition, the periosteum “spindle-” shaped cells had significantly higher cell surface area and cell thickness (unpaired Student's *t*-test, *P* < 0.0001) compared to BM cells. Overall, these data indicated that periosteal cells MSCs appear to have a larger cell volume than their BM counterparts.

**Figure 4:**
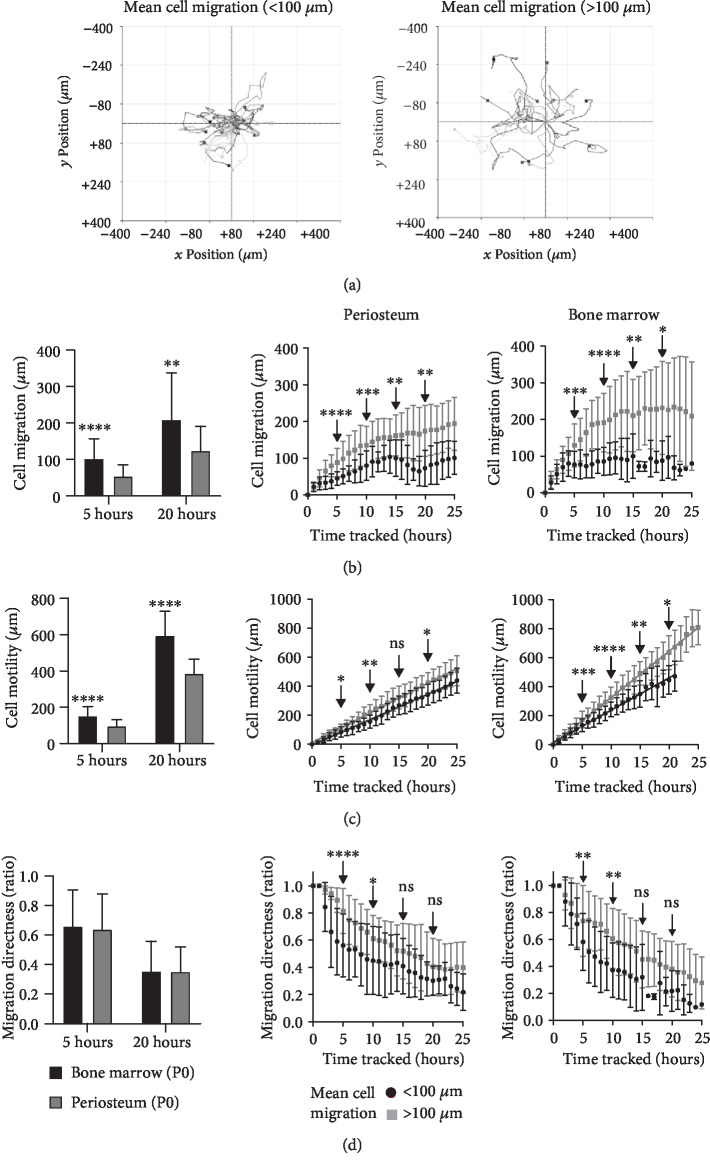
Live cell tracking of migration patterns of periosteum and bone marrow cultures. (a) Cell migration map of individually tracked cells with </>100 *μ*m mean cell migration, multiple cells represented. (b) Quantification of cell migration, (c) cell motility, and (d) cell migration directness for periosteum and bone marrow cultures over time. (A) Quantified at 5 and 20 hours; (B, C) individually tracked cells were split into </>100 *μ*m mean cell migration for periosteum (B) and bone marrow (C). Unpaired Student's *t*-test, *P* < 0.05, ^∗∗∗∗^*P* < 0.0001, ^∗∗∗^*P* < 0.001, ^∗∗^*P* < 0.005, and ^∗^*P* < 0.05, ns: not significant.

The cell migration and movement patterns of periosteal and BM MSCs were assessed by live tracking of individual cells. Maps of cell movement (Figure 4(a)) revealed visual differences in MSC migration patterns of individual cells, whereby some cells were relatively nonmigratory, remaining in the same area, and other cells were seen to migrate across the field of view. “Cell migration” refers to the displacement of a tracked cell from the first measured coordinates, in comparison to “cell motility” which refers to the cumulative distance travelled at each time point. From three fields of view per culture, the whole cell population was quantified over 25 hours and mean cell migration was calculated as well as split into the migratory (>100 *μ*m cell migration) or the nonmigratory (<100 *μ*m cell migration) cells (Figure 4(a)).

To track changes in migration patterns over time, mean cell migration after 5 and 20 hours of cell tracking was compared. BM MSCs showed significantly higher mean cell migration (unpaired Student's *t*-test, *P* < 0.0001 (5 hours), *P* = 0.001 (20 hours)) at 5 hours (102.6 ± 54.6 *μ*m (BM), 53.5 ± 32.6 *μ*m (periosteum)) and 20 hours (209.1 ± 129.2 *μ*m (BM) and 123.6 ± 67.6 *μ*m (periosteum)) (Figure 4(b)). A similar trend was seen with cell motility, where BM MSCs were significantly more motile over time (unpaired Student's *t*-test, *P* < 0.05). At 5 hours, total cell motility (distance travelled) was 151.4 ± 53.6 *μ*m (BM) compared to 95.6 ± 35.0 *μ*m (periosteum), and by 20 hours, this difference had increased to 594 ± 136.0 *μ*m (BM) and 384.2 ± 81.5 *μ*m (periosteum) (Figure 4(c)).

The proportion of nonmigratory to migratory MSCs within the periosteum and BM cultures was 42% (*n* = 45) and 63% (*n* = 94), respectively, suggestive that there is a slightly lower proportion of cells classified as nonmigratory within periosteum cultures (Table 3). When comparing nonmigratory to migratory MSCs over time, significant increases in cell migration or displacement away from the initial tracked point were seen from 5 hours onwards between the migratory and nonmigratory cells for both culture types (unpaired Student's *t*-test, *P* < 0.05) (Figure 4(b)). Similar trends were seen, where cell migration steadily increased in a linear fashion and then started to plateau; however, while the plateau was seen at approximately 8 hours (>100 *μ*m) and 5 hours (<100 *μ*m) for BM cultures, this was extended to about 15 hours (<100 *μ*m) or not well defined for >100 *μ*m within periosteum cultures (Figure 4(b)).

Based on this data, it could be concluded that cell migration was not only “faster” in BM MSCs compared to their periosteum counterparts but also “further away” from the tracked start point (approximately 190 *μ*m (BM) vs. 150 *μ*m (periosteum)) (Figure 4(b)), indicating that the speed of cell movement was greater in BM cultures.

Significant increases in cell motility or distance (rather than displacement) migrated over time were seen with the migratory cells (>100 *μ*m), compared to the nonmigratory cells (<100 *μ*m) from 5 hours onwards (unpaired Student's *t*-test, *P* < 0.005), irrespective of culture type (Figure 4(c)). Cell motility was linear, irrespective of either class of migration, with *r*^2^ values of >0.994 for both culture types. Therefore, cell speed (*μ*m/h) was calculated from the gradient of the cell motility graphs (Figure 4(c)); as expected, the speed of the migratory cells was higher than the nonmigratory cells (Table 3). Furthermore, BM MSCs were shown to migrate at either 22.3 *μ*m/h (<100 *μ*m) or 32.0 *μ*m/h (>100 *μ*m), confirming that BM MSCs move with greater speed than periosteum MSCs (17.5 *μ*m/h (<100 *μ*m), 20.4 *μ*m/h (>100 *μ*m)) (Table 3).

Migration directness is a ratio of migration (displacement) to motility (distance), where 1 refers to movement in a straight line and 0 to a cell moving completely randomly. This parameter can be used to calculate whether cells migrate randomly or with apparent “purpose.” In contrast to the previous parameters, no significant differences were seen between the MSC culture types over time (Figure 4(d)). Cell migration directness reduced over time, with the nonmigratory cells showing significantly reduced cell directness at 5 and 10 hours for both MSC types (unpaired Student's *t*-test, *P* < 0.05); however, after this, differences were not seen, suggestive that with time both MSC types migrated in a more random fashion.

Overall, these data provided new insights into BM and periosteum MSC behaviour in culture. Smaller volumes of BM MSCs could facilitate their faster migration capacities, which in turn could in part explain their ability to form larger colonies, compared to periosteal MSCs, in standard CFU-F assays.

### 3.4. MSC Differentiation Capacity

**Figure 5:**
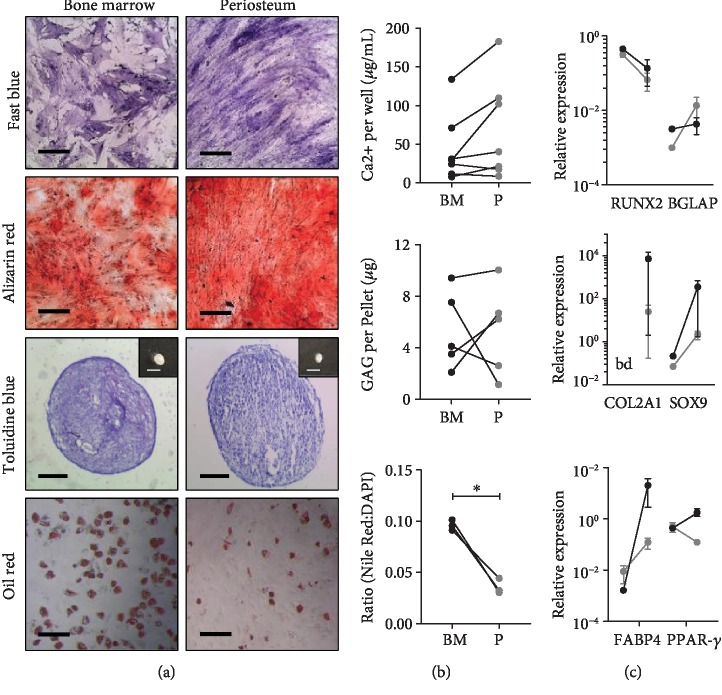
Trilineage differentiation assays, comparing donor-matched periosteum and bone marrow MSCs. Each assay was assessed using (a) histology staining, (b) quantitative assays, and (c) qPCR for specific markers of differentiation. Osteogenic assays stained for alkaline phosphatase (fast blue) and calcium deposition (alizarin red), with quantified Ca^2+^ content and assessment of runt-related transcription factor 2 (*RUNX2*) and bone gamma-carboxyglutamate (gla) protein (*BGLAP*) expression levels. Chondrogenic assay macro images (insert) stained for glycosaminoglycans (GAG) (toluidine blue), quantified GAG content and collagen, type 2, alpha 1 (*COL2A1*), and SRY- (sex-determining region Y-) box 9 (*Sox9*) expression levels. Adipogenic assays stained for fat deposition (oil red), quantified fat deposition (Nile red) and nuclei content (DAPI), and fatty acid-binding protein 4 (*FABP4*) and peroxisome proliferator-activated receptor-gamma (*PPAR-γ*) expression levels. Wilcoxon signed-rank test was carried out, ^∗^*P* < 0.05. Scale bars represent 500 *μ*m (fast blue, alizarin red, and oil red), 200 *μ*m (macro insert), and 200 *μ*m (toluidine blue). BM: bone marrow, P: periosteum, bd: below detection.

Quantitative trilineage differentiation assays were carried out on donor-matched cultures in addition to qPCR for lineage-specific markers comparing day 0 (before differentiation) and day 21 (following three weeks of differentiation induction). Osteogenic cultures were stained for ALP after two weeks and then for calcium deposition after three weeks, where visually similar staining could be seen for both culture types, with a tendency for visually higher confluency in the periosteum cultures (Figure 5(b)). Calcium content was quantified (*n* = 7) at three weeks and was found to be slightly higher (but not significant, Wilcoxon signed-rank test, *P* = 0.08) in periosteum than BM cultures. Of note, there was high donor variation that was not shown to correlate with donor age (*r*^2^ value, 0.17 (BM), 0.22 (periosteum)). qPCR showed reduction in the early osteogenic marker, *RUNX2* expression, but increases in the late osteogenic marker *BGLAP* expression at day 21 (*n* = 3) for both culture types, as expected.

In chondrogenic conditions, after three weeks, pellets of similar sizes were seen to form for periosteum and BM cultures and staining for GAG (toluidine blue) was confirmed for both (Figure 5(b)). Following quantification of GAG content, no significant differences were seen between periosteum and BM pellets (Wilcoxon signed-rank test, *n* = 5, *P* = 0.99) (Figure 5(b)). As with osteogenesis, there was high donor variability; however, donor age was seen to negatively affect GAG production for BM cultures (*r*^2^ = 0.77), but not periosteum cultures (*r*^2^ = 0.22). Chondrogenic differentiation markers, *COL2A1* and *SOX9*, were increased in both periosteum and BM pellets by day 21 (Figure 5(b)).

Noticeably, greater fat deposition was seen in BM cultures compared to periosteum, following three weeks of adipogenic induction and oil red staining (*n* = 6) (Figure 5(b)). Quantification of fat deposition (Nile red) and DNA (DAPI) levels revealed that BM cultures had higher fat per cell content (Wilcoxon signed-rank test, *P* < 0.05). *FABP4* was seen to increase in both culture types following adipogenic induction (Figure 5(b)); however, *PPAR-γ* increased in BM cultures but reduced in periosteum cultures.

## 4. Discussion

In this study, the potential for the utilisation of periosteum-derived MSCs as a source of MSCs during bone defect surgical repair was investigated. Currently, there is a lack of direct comparisons of MSCs derived from human periosteum and BMA, especially when donor matched [24, 35]. Here, for the first time, donor-matched comparisons of “clinically accessible” periosteum (from within the surgical opening of a fracture site) were compared to iliac crest BMA, a supply of MSCs currently used in surgery [36].

Both MSC sources were compared histologically to locate CD271+ candidate MSCs within each tissue [21, 37–39]. The cambium layer of periosteum has been historically described as a rich source of osteoprogenitors, whereas the fibrous layer is thought to be less cellular [16, 17, 40, 41]. However, this study has shown the presence of CD271+ cells in human samples located throughout the outer fibrous layer. These cells were localised surrounding blood vessels throughout the fibrous layer; therefore, even though the cambium layer was often not harvested, these data indicated that “clinically accessible” human periosteum could still contain a supply of MSCs.

CFU-F assays are the gold standard for MSC quantification in BM aspirates or solid connective tissues enzymatically processed to release viable cells [42]. Despite this, very few studies have so far quantified human periosteum MSC frequency [20, 42] and none of them compared it to donor-matched BMA. Our data on the 2500-fold higher frequency of MSCs present within “clinically accessible” periosteum samples, which most commonly do not include the cambium layer, present periosteum as a viable alternative to BM aspirates with high and consistent supply of MSCs. Of note, the MSC counts within BM samples (0.0008%) in this study were lower compared to the literature, which estimates 0.001-0.01% MSC [43]. This could be due to the fact that most of the BMA in our study were collected in large volumes (>50 mL) prior to concentration to generate BMAC, and it is known to result in BM dilution with peripheral blood [21, 32, 43]. Nevertheless, even at the highest MSC content for BMA (0.0027%) within this study, the donor-matched periosteum had >1500-fold more MSCs, thus confirming the superiority of periosteum samples with respect to their MSC content.

Until recently, CFU-F colony size measurements have not been routinely performed in MSC tissue comparison studies; however, these data can provide valuable information on MSC heterogeneity within the tissues under investigation. When this was carried out on BM aspirates, MSC subpopulations with different sizes and densities have been found indicating a high degree of MSC heterogeneity, which in one study was linked to donor age [22] but could be also related to different MSC topographies [44, 45]. Here, periosteum colonies were significantly smaller (half in size), and more homogenous than donor-matched BM counterparts, which aids in the predictability of periosteum MSC outcomes over BM MSCs.

In order to underpin the differences seen in colony formation between periosteum and BM MSC cultures, live cell imaging of early *in vitro* (2 weeks in culture) MSCs was carried out. Phase holographic imaging was utilised as a noninvasive, unlabeled high throughput method for quantifying cellular morphology and live cell tracking [46–48]. MSC morphology in both MSC sources was variable over time, changing hour by hour, with approximately 98% of the cells measured retaining the classical “spindle” shape, and the remaining 2% of the measured cells were retracted into spheres, undergoing division. Similar proportions of dividing cells seen in these experiments are consistent with our data on similar growth rates between paired periosteum and BM cultures prior to passage zero. Based on these and our CFU-F data, we therefore hypothesised that smaller periosteum colony sizes could be due to their reduced cell area and/or poorer ability for migration rather than their reduced proliferation.

When individual cells were analysed over a 24-hour period, it was observed that as MSCs proliferate and occupy more surface, their migration plateaus and cell directness becomes more random, for both cell types. Indeed, periosteum MSCs were less migratory and moving at slower speeds than BM MSCs. This was also true when cells were divided into two subpopulations, nonmigratory and migratory, based on chosen arbitrary cut-off point of 100 *μ*m. The speed at which MSCs migrate was also quantified; for the first time, periosteum MSCs were shown to migrate at 17.5 *μ*m/h (nonmigratory) and 20.4 *μ*m/h (migratory), which was slower than their BM MSC counterparts at 22.3 *μ*m/h (nonmigratory) and 30.0 *μ*m/h (migratory). BM MSC migration speed within a collagen type I gel has been quantified previously in one study (15 *μ*m/h), which could reflect that migration speed is influenced by the substrate on which MSCs are in contact with [49]. This was also shown by Salam et al. [50], whereby fibrinogen matrix concentration was shown to inversely affect BM MSC migration, through reduction in pore size and changes in substrate stiffness. These characteristics may help to develop more rational approaches for bone scaffold design for repopulation by periosteal cells, for example, their optimal porosity. Related to this, a recent study utilised holographic imaging to assess growth and differentiation of BM MSCs onto a glass and a titanium oxide (TiO_2_), showing favorable cell adhesion and spreading on the TiO_2_ coating [51], highlighting the potential of this technology in the tissue engineering field. Future work would aim at increasing donor numbers to assess donor variability and impact of donor age, as well as a more precise study of how cell confluence affects individual cells' behaviours.

With respect to cell size analysis, periosteum MSC cell area was 20% larger compared to donor-matched BM cells. This confirmed that despite higher cumulative PDs accrued by BM MSCs, they were not approaching senescence (senescent cells are larger) [52], which is consistent with our observations of similar proportions of dividing cells for both MSC types. Although this investigation was limited to a single pair of donor-matched cultures, the available evidence points towards slower motility of early-culture periosteal cells compared to BM cells in our experimental conditions, rather than their lower proliferation or smaller cell area. A larger cell surface area of periosteum MSCs could affect the traction forces required to overcome fractional and adhesive resistances required to allow for cell movement [53]. Confluence is another factor that can affect cell velocity or migration; however, this did not appear to influence the seen differences in this study, thus other factors must be at play.

We have shown that differing tissue sources of MSCs, from the same donor and grown *in vitro* for the same time period, are potentially interacting with the same substrate (in this case tissue culture plastic) in different ways, thus resulting in changes in cell surface area, migration distance, and velocity. It is known that migration of spindle-shaped cells, like MSCs, is dependent on the formation focal adhesions with actin filaments that mechanically link the extracellular matrix to the cytoskeleton and contractile stress fibres [53]. Levels of integrins and cytoskeleton within MSCs have previously been linked to influencing MSC differentiation, whereby increased focal adhesions and a stiff spread cytoskeleton appear to influence osteogenesis [54]. Goessler et al. [55] showed that adipose tissue MSCs and BM MSCs grown *in vitro* express different levels of various integrins, which influence focal adhesion formation. However, little is known about differences in integrin and focal adhesion levels between periosteum and BM MSCs; future developments to this work would involve ascertaining whether there are inherent differences in the cytoskeleton and focal adhesion formation.

For periosteum to be utilised as a source of MSCs for fracture healing, osteogenic and chondrogenic capacities are of particular importance [56, 57]. While osteogenic and chondrogenic differentiation capacities of periosteum MSC cultures were shown to be similar to donor-matched BM MSCs, adipogenic differentiation showed clear differences, whereby periosteal adipogenic potential was significantly lower than donor-matched BM MSCs. Differences could be partially explained through a reduction in *PPAR-γ* levels, a molecule critical to initiating adipocyte differentiation [58], in day 21 periosteum MSCs, whereas in BM MSCs, it was upregulated, consistent with previous studies [24, 31]. Mastoid periosteum MSC clones showed variation in trilineage differentiation capacity, whereby nearly all clones showed osteochondral capacity, whereas only 53% were considered to be adipogenic [59]. Based on this and our data, it could be suggested that periosteum MSCs have a preferential commitment to osteochondral differentiation, needed for fracture repair.

As with harvesting of bone autograft for filling of critical size bone defects, a key point to consider with advocating for the use of periosteum is creating a minimal donor site morbidity. Currently, free vascularised corticoperiosteal bone grafts, where the periosteum and underlying cortical graft bone can be 4 cm^2^, are harvested from the medial femoral condyle, to wrap around a defect site [60–63]. The femoral condyle is found to consolidate well following the harvesting process [60, 61]. Additionally, proof-of-concept studies in critical size bone defect sheep models have shown maximal defect bridging, where autologous periosteal strips were used, harvested with a periosteal elevator, thus removing the cambium layer [64, 65]. Together, this suggests that harvesting small graft(s) with a scalpel of the fibrous layer of periosteum as in this study, leaving the cambium mainly intact, would not create a donor site morbidity. This would allow for periosteum as a proliferative “MSC-rich” source to be transplanted into or around defect areas for the treatment of complex fracture or critical size bone defects, as a conjunct with other healing stimulating factors and cell sources that are currently in use.

## 5. Conclusions

“Clinically accessible” samples of long bone periosteum, a vital component of bone fracture repair has been shown to be a rich source of highly proliferative MSCs, compared to the current “gold-standard” BMA, with lower adipogenic but similar osteochondral potential. A novel live cell tracking technique permitted extensive quantification of morphological and migratory characteristics of periosteal MSCs that can be used to inform the development of novel bone graft substitutes to be repopulated by these cells. Further investigation into “minimally manipulated” periosteum samples, for example, periosteal micrografts, is needed for future clinical translation of this tissue source for use during single surgical procedure.

## Figures and Tables

**Table 1:** Breakdown of patient demographics, with respect to location of periosteum retrieval and time since initial injury for histological analysis and cellular work.

Sex & age (years)		Harvested from	Time since injury (weeks)	Previous surgeries	Surgery carried out
Histology	M, 23^∗^	Femur	57	2	IMN
	M, 35^∗^	Humerus	108	None	Bone graft with BMAC
	M, 47	Femur	19	None	RIA graft
	F, 74^∗^	Femur	49	2	IMN, bone graft
	F, 80	Femur	17	2	2^nd^ stage Masquelet

Cellular work	M, 17	Femur	65	2	IMN
	M, 44	Tibia	11	1	IMN
	F, 49	Femur	155	1	IMN
	M, 49	Femur	12	1	Plate fixation
	M, 55	Femur	41	1	2^nd^ stage Masquelet
	M, 58	Tibia	71	1	Bone graft
	M, 59	Femur	127	2	Locking plate

M: male, F: female, none: sample harvested during first orthopaedic surgery, IMN: intramedullary nailing, BMAC: bone marrow aspirate concentrate, RIA: reamer irrigator aspirator, ^∗^sample also used for cellular work.

**Table 2:** Summary of cell morphology of periosteum and bone marrow cultures. Periosteum (P) vs. bone marrow (BM), unpaired Student's *t*-test, ^∗∗∗∗^*P* < 0.0001.

Cell morphology characteristic		Number of cells measured	Average cell thickness (*μ*m)	Cell surface area (*μ*m^2^)
Dividing	P	51	6.1±2.5^∗∗∗∗^	497 ± 172
	BM	23	3.4 ± 0.6	493 ± 126

Spindle	P	2438	1.4±0.3^∗∗∗∗^	827±414^∗∗∗∗^
	BM	1061	1.3 ± 0.3	668 ± 297

**Table 3:** Summary of confluency, cell migration, and cell motility speed—split into nonmigratory and migratory cells, following cell tracking of periosteum and bone marrow MSC cultures.

Passage 0 MSC culture	Confluency (% cell coverage)		Number of cells split by cell migration (%)		Cell motility (*μ*m/h)	
	0 hours	25 hours	<100 *μ*m	>100 *μ*m	<100 *μ*m	>100 *μ*m
Periosteum	5.0 ± 1.2	9.9 ± 3.3	42.2 (*n* = 55)	57.8 (*n* = 26)	17.5	20.4
Bone marrow	0.9 ± 0.3	2.7 ± 0.6	62.8 (*n* = 59)	37.2 (*n* = 35)	22.3	32.0

## Data Availability

The data used to support the findings of this study are available from the corresponding author upon request.

## Supplementary Materials

Supplementary Materials Details of the antibodies used to phenotype MSCs with flow cytometry can be found in Supplementary Table 1.
