# Supplementary material for: Colony Formation, Migratory, and Differentiation Characteristics of Multipotential Stromal Cells (MSCs) from “Clinically Accessible” Human Periosteum Compared to Donor-Matched Bone Marrow MSCs
Source: Stem Cells Int. 2019 Nov 21;2019:6074245. doi: 10.1155/2019/6074245 (PMC6906873; doi:10.1155/2019/6074245)
Supplement: Supplementary Materials — Details of the antibodies used to phenotype MSCs with flow cytometry can be found in Supplementary Table 1. [file 6074245.f1.pdf]

Supplementary Table 1: Details of antibodies used for flow cytometry phenotyping of MSCs.

Antibody		Clone	Fluorochrome	Volume (μL)	Manufacturer
Positive MSC Markers	CD73	AD2	PE	5.0	Miltenyi Biotec
	CD90	SE10	PECy7	2.5	BD BioSciences
	CD105	SN6/REA794	PE	10.0	Miltenyi Biotec
Isotype Controls	IgG1	IS11-12E4.23.30	APC	5.0	Miltenyi Biotec
	IgG1	MOPC-21	FITC	5.0	BD BioSciences
	IgG1	MOPC-21	PE	5.0	BD BioSciences
	IgG1	MOPC-21	PECy7	2.5	BD BioSciences
	IgG1	MOPC-21	VioGreen	2.0	Miltenyi Biotec
	IgG2a	MCA1210	FITC	5.0	BioRad
Negative MSC Markers	CD14	TUK4	FITC	5.0	Miltenyi Biotec
	CD19	HIB19	PE	10.0	BD BioSciences
	CD34	581	APC	10.0	BD BioSciences
	CD45	HI30	PECy7	2.5	BD BioSciences
	HLA-DR	REA805	VioGreen	2.0	Miltenyi Biotec
